# Comprehensive assessment of the quality of *Salmonella* whole genome sequence data available in public sequence databases using the *Salmonella in silico* Typing Resource (SISTR)

**DOI:** 10.1099/mgen.0.000151

**Published:** 2018-01-17

**Authors:** James Robertson, Catherine Yoshida, Peter Kruczkiewicz, Celine Nadon, Anil Nichani, Eduardo N. Taboada, John Howard Eagles Nash

**Affiliations:** ^1^​ National Microbiology Laboratory, Public Health Agency of Canada, Guelph, ON, Canada; ^2^​ National Microbiology Laboratory, Public Health Agency of Canada, Winnipeg, MB, Canada; ^3^​ National Microbiology Laboratory, Public Health Agency of Canada, Lethbridge, AB, Canada; ^4^​ National Microbiology Laboratory, Public Health Agency of Canada, Toronto, ON, Canada

**Keywords:** *Salmonella*, Public Health, whole genome sequencing, serotyping, surveillance, phenotype prediction

## Abstract

Public health and food safety institutions around the world are adopting whole genome sequencing (WGS) to replace conventional methods for characterizing *Salmonella* for use in surveillance and outbreak response. Falling costs and increased throughput of WGS have resulted in an explosion of data, but questions remain as to the reliability and robustness of the data. Due to the critical importance of serovar information to public health, it is essential to have reliable serovar assignments available for all of the *Salmonella* records. The current study used a systematic assessment and curation of all *Salmonella* in the sequence read archive (SRA) to assess the state of the data and their utility. A total of 67 758 genomes were assembled *de novo* and quality-assessed for their assembly metrics as well as species and serovar assignments. A total of 42 400 genomes passed all of the quality criteria but 30.16 % of genomes were deposited without serotype information. These data were used to compare the concordance of reported and predicted serovars for two *in silico* prediction tools, multi-locus sequence typing (MLST) and the *Salmonella in silico* Typing Resource (SISTR), which produced predictions that were fully concordant with 87.51 and 91.91 % of the tested isolates, respectively. Concordance of *in silico* predictions increased when serovar variants were grouped together, 89.25 % for MLST and 94.98 % for SISTR. This study represents the first large-scale validation of serovar information in public genomes and provides a large validated set of genomes, which can be used to benchmark new bioinformatics tools.

## Data Summary

Genome metadata and analysis for publicly available genomes have been deposited in Figshare; DOI: 10.6084/m9.figshare.5464396 (https://figshare.com/s/fc4adaac52a678d92c8b) referred to as Data S1.

Snapshots of MLST data from http://mlst.warwick.ac.uk/ have been deposited in Figshare; DOI: 10.6084/m9.figshare.5464396 (https://figshare.com/s/fc4adaac52a678d92c8b) referred to as Data S2.

## Introduction

*Salmonella* is a priority pathogen causing significant morbidity and mortality around the world; a significant proportion of the estimated 230 000 deaths caused annually by bacterial diarrhoeal diseases are due to *Salmonella* [1]. Surveillance systems designed to detect, solve and help prevent the occurrence of salmonellosis depend on reliable, standardized characterization of *Salmonella*. For more than 100 years, the gold standard for classifying *Salmonella* has been serotyping, with additional subtyping applied as required for higher level resolution [2, 3]. *Salmonella* isolates are grouped into serovars using the White–Kauffman–Le Minor (WKL) scheme on the basis of reaction of antisera to somatic (O) and flagellar (H) antigens [4]. Public health surveillance infrastructure for *Salmonella* is still based on serological nomenclature despite increasing adoption of whole genome sequencing (WGS). Current laboratory tests generate one result per assay but the benefit of WGS is that it is a single diagnostic test which provides the information necessary to run any number of additional tests as the need arises [5]. PulseNet International, the global network responsible for standardized surveillance and outbreak response, has recently promised the transition of all 86 member countries to WGS, with the ultimate vision of having all of the data being made public [6]. Several major institutions around the world have already completed this transition, including the US and the UK [7, 8]. As more laboratories utilize *Salmonella* WGS within public health, it is important to maintain serovar designation to minimize disruptions to ongoing public health surveillance activities.

Due to the problems associated with user-submitted taxonomy, GenBank has implemented pipelines for validating microbial species taxonomic assignments to reduce the negative impact misidentified genomes present to the community [9]. Mechanisms are already in place to suppress incorrect or low-quality data within the nucleotide repository but the raw data within the sequence read archive (SRA) currently do not undergo the same level of scrutiny. Genomic data from WGS provide an unprecedented level of information about an organism but the billions of bases available mean little without the contextual information of the isolate. Both the sequence information and the metadata must be trustworthy for correct conclusions to be drawn from any analyses. At present, there are more than 70 000 *Salmonella* WGS runs available in the SRA with a continuing growth of ~2000 runs each month. Issues with the quality of nucleotide data in public repositories including contamination, chimeras and taxonomic misidentification have been the subject of multiple publications and it can be considered a certainty that any public repository will contain errors [10–16]. The *Salmonella in silico* Typing Resource (SISTR) was developed to accurately predict the serovar of an isolate based on draft genome assemblies using O- and H-antigen’s genetic determinants alongside a core genome multi-locus sequence typing (cgMLST) to improve discrimination between related serovars [17]. A rigorous validation of the SISTR platform was performed and due to the accuracy of the system, it is being implemented within the Public Health Agency of Canada alongside WGS as a replacement for serological typing of *Salmonella* isolates [2].

To our knowledge there has not been a comprehensive survey of all of the publicly available SRA data available for *Salmonella* or similarly sequenced organisms. Since serovar information is one of the most critical pieces of contextual information for *Salmonella* provided in submissions to the public WGS repositories, we wanted to identify and evaluate the types of errors present utilizing SISTR, and provide recommendations to avoid these errors in future submissions. We have identified a large set of genomes for which the serovar information has been validated and sample metadata have been standardized. This validated dataset is being provided as a resource to the *Salmonella* research community.

**Impact Statement**

*Salmonella* is a major source of bacterial food infection worldwide. Public health laboratories are increasingly adopting whole genome sequencing (WGS) methodologies to characterize and track *Salmonella* outbreaks. Many public health laboratories are depositing raw WGS data associated with outbreaks of *Salmonella*, as well as data associated with surveillance activities into public repositories. For these data to be of use in comparative genomics and epidemiological studies in a public health context, it is important that the metadata associated with these sequences (e.g. data/time of collection, geography, associated host of environment and in particular its serotype) be as accurate as possible. We previously reported an *in silico* platform for serotyping WGS data and found high concordance between reported and predicted serotypes [2, 17]. We systematically assessed the accuracy of serotype information in the public repositories using the *Salmonella in silico* Typing Resource (SISTR), and where possible corrected the serotype assignments. We assembled the raw WGS data for 67 758 isolates from the sequence read archive and produced serotype predictions. The corrected data will be useful in public health meta-analyses regarding *Salmonella*, and this large trusted dataset will be useful for the quality control of data deposited into public repositories.

## Methods

### Data standardization

Sample information was retrieved for each *Salmonella* Illumina SRA run available as of January 2017. Five highly important metadata fields were selected for manual curation to standardize the contained terms to improve the value of the sequence record: Serovar, Collection Date, Host, Isolation Source and Collection Location. All original information for each record was maintained but a curated field was created in Data S1 (available in the online version of this article) to indicate the curated value for the field. Serovar information was validated against the WKL scheme to standardize serovar synonyms and correct variations and misspellings. Serovar variants were separated into a new field. The serovar field was blanked from further analysis, if the information was not a valid serovar within the WKL scheme. Collection dates were standardized to the format YYYY-MM-DD with a collection date accuracy field to indicate ambiguity in the collection date. For example, if the record only contained the year of collection the collection date would be set to Jul-02 of that year and the collection date accuracy would be set to +/−182 days. Geographical locations were standardized to Country and State/Province according to an in-house reference list.

Host and isolation sources were highly heterogeneous in their content and many submitters used the fields interchangeably. To aid in the use of the Isolation Source information, we created categories that could be assigned to the records. Manual review of the sample information yielded six primary classifications for records: Animal, Animal-Feed, Human, Plant, Prepared Food Product and Environmental. Secondary categories were developed according to the most frequently occurring categories, which could be used to group records. For example, poultry is a secondary classification, which could be applied to multiple different sample types. Instead of a Host field, we created the field Associated Taxa, which can be used to indicate a relationship between a sample and an organism. This field uses the Linnaean nomenclature with the level of specificity available. If there is ambiguity in the taxonomic assignment, then a broader taxonomic division is used which would reflect the source. This allows users to select records found with an organism, which might not be the true host. For example, cow cheese is a Prepared Food Product with *Bos taurus* as the associated taxon.

### Retrieval and assembly of genomes

Raw Illumina paired-end reads were downloaded from the SRA and assembled using the pipeline described below. Reads were corrected using Lighter v. 1.1.1 (https://github.com/mourisl/Lighter). Overlapping read pairs were joined using FLASH v. 1.2.11 (https://ccb.jhu.edu/software/FLASH/) and assembled using SPAdes v. 3.6.2 (http://spades.bioinf.spbau.ru/) using the joined reads along with the paired-end reads with the *careful* option specified. The default option for SPAdes v. 3.6.2 includes error correction using Bayes Hammer. Assembled genomes were also assessed for contamination using Kraken v. 0.10.5-beta (https://ccb.jhu.edu/software/kraken/) and the reference database set to be MiniKraken DB (8 December 2014). A custom script was used to take the Kraken output and calculate the percentage abundance of each taxon in a given assembly (https://github.com/jrober84/sra-manuscript). The Kraken summary is based at a contig level and as such only considers sequences which contributed to the overall assembly.

### Sequence analysis of genomes

Assembled genomes were uploaded to SISTR (https://lfz.corefacility.ca/sistr-app/) via the application programming interface (API) and the resulting serovar predictions were compared to the reported serovar on the NCBI record. Algorithmic details for the SISTR are as previously described [17]. The cgMLST schema and SISTR tool code base can be accessed at https://bitbucket.org/peterk87/sistr_backend/wiki/Home. The raw NCBI metadata were processed and normalized to standardize serovar names or antigenic formula where possible through manual curation. Deprecated serovar names were converted to their valid name in the WKL to ensure consistent naming of serovars. SISTR does not report serovar variants, which require additional testing so the comparisons here separate the variant status from the serovar call. The predicted serovar was compared to the reported serovar and the results were categorized into five types: Type 0, Full match; Type 1, Incorrect reported serovar; Type 2, Serovar variant detected; Type 3, Incorrect predicted serovar; Type 4, Untypeable. A genome was categorized as Type 1 if the reported serovar was not supported by antigenic calls, MLST or cgMLST. Genomes were assigned to Type 2 in cases where the reported serovar was biphasic but only one flagellar gene was found. Type 3 errors were due to an algorithmic issue, which resulted in a prediction that is not supported by the genetic evidence. In these cases, examination of the antigenic genes, cgMLST and MLST data supported the original reported designation. Type 4 errors were assigned to a genome when a given approach was unable to produce a serovar call. These types of errors are largely due to missing antigenic factors where we were unable to confirm the reported serotype. SISTR incorporates MLST into its outputs but it does not provide fuzzy matching in the case of incomplete MLST calls, so genomes were also assigned to sequence types (STs) using the MLST v. 2.0 tool (https://github.com/tseemann/mlst). Partial or imperfect matching alleles are reported and individual allele calls are available (Data S1).

Draft *de novo* genomes were used to build core genome single nucleotide (cgSNP) trees using the Parsnp aligner v. 1.2 [18] with default parameters, allowing for automatic recruitment of the reference sequence and requiring that all genomes be included for the analysis. Trees were then examined in the Gingr v. 1.2 2 [18] alignment viewer to examine the cgSNPs. The exported tree was annotated using FigTree v. 1.4 (http://tree.bio.ed.ac.uk/software/figtree/). Flagellar protein sequences were extracted from genomes using the blast results produced by SISTR and were then aligned using the web version of Clustal Omega (http://www.ebi.ac.uk/Tools/msa/clustalo/) and invariant 5′/3′ ends were removed to highlight the variable core region of the protein.

## Results

### Genome assemblies QA/QC

Using the data available in Data S1 for 67 758 genomes, the distribution was determined for each quality metric. Using this distribution information we developed criteria for filtering genomes as ‘low quality’ or ‘atypical’ so that they would be removed from any downstream analyses. Summaries of the number of genomes failing a particular filter are shown in Table 1. A total of 1264 genome assemblies had >10 % non-*Salmonella* DNA sequences present based on analysis using Kraken (Table 1). *Salmonella* isolates generally have a genome size between 4.3 and 5.3 Mb, with an average size of 4.7 Mb (Data S1). Allowing for some variance in accessory gene content, an assembly was flagged as ‘atypical’ if it had a size outside the range of 4–6 Mb. Overall, the majority of assemblies fit into this range with the exception of 1937 genomes (Table 1).

**Table 1.** Draft genome assemblies were put through each quality control filter in parallel and the numbers of genomes failing at each filter are listed above The unique list of genomes that failed one or more filters was found to be 25 358 of the 67 758 genomes used as input. Genomes with more than 10 % of their assembled bases from non-*Salmonella* failed the contamination check. Assemblies with a size <4 or >6 Mb failed the size criteria. Assemblies were filtered if they met any of these conditions: with >500 contigs, largest contig <100 kb, more than 1 % ambiguous bases, an N50 <50 kb. A further reason for exclusions was if the assembly did not have ≥300 cgMLST genes. If the GC content of the genome was <50 % or >54 % it was excluded. Finally, if the genome was not associated with a serovar in the SRA it was not analysed further in the comparison of the *in silic*o tools. N50, the value where 50 % of an assembly is made up of contigs equal to or greater than that value.

Reason for exclusion	Count	Percentage
Contamination	1267	5.00
Genome size	1937	7.64
Number of contigs	1094	4.31
Largest contig	674	2.66
Ambiguous	299	1.18
Missing O-/H-antigen	695	2.74
cgMLST allele count	1443	5.69
N50	2087	8.23
GC content	1017	4.01
Missing serovar assignment	20 446	80.63
Cumulative fail	25 358	

**Fig. 1.**
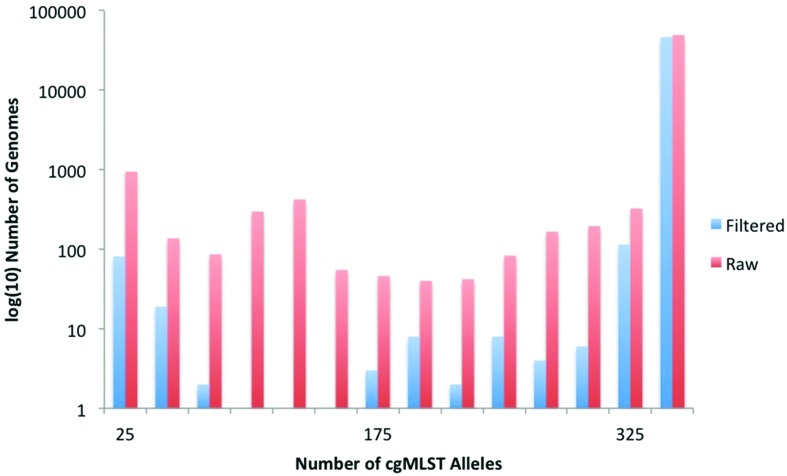
Draft genome assemblies were examined through SISTR to determine the number of the 330 cgMLST genes present in each assembly. A histogram of the frequency of cgMLST abundance in genomes was determined for the entire dataset (67 758 genomes) without any quality control. The analysis was then repeated on filtered genomes that passed the sequence quality filters with the exception of the requirement for >320 cgMLST genes.

SISTR’s conservative 330 cgMLST scheme is readily retrievable from assemblies, with 92 % of all genomes possessing the full 330 gene complement and ~95 % of assemblies possessing >300 complete cgMLST loci (Fig. 1), which rise to ~96 % and ~98 %, respectively, after adjusting for contamination and other genome quality metrics (Fig. 1). We selected a threshold of 320 cgMLST loci identified in the genome in order to consider it for serovar comparisons, which removed 1443 genomes from the dataset (Table 1). A final criterion for inclusion into the dataset was the presence of at least one flagellar or O factor antigen gene, which removed a further 695 isolates (Table 1). The filter with the greatest impact on the dataset was the requirement for a serovar to be specified in the record, with 20 446 genomes failing this check (Table 1). Those genomes that met our QC criteria were assigned predicted serovars using SISTR and were included in the final validated dataset (Data S1). SISTR predictions have been shown previously to be highly concordant with traditional serotyping up to 94.8 % when appropriate quality criteria are in place [2, 17].

### Composition of SRA *Salmonella* entries

**Fig. 2.**
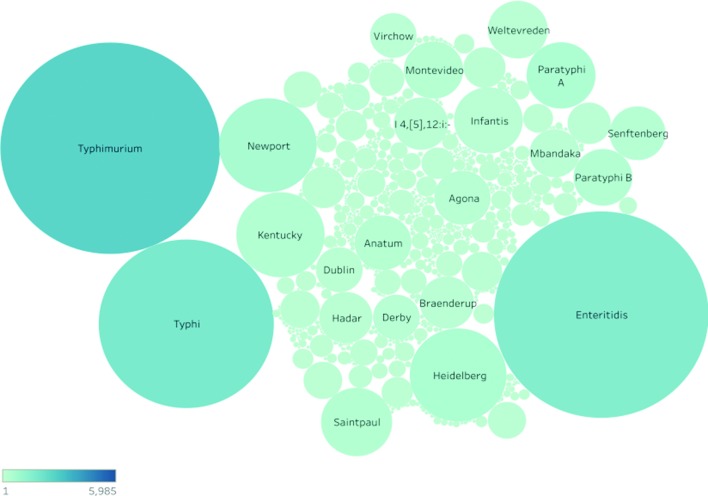
Bubble graph of the intra-serovar diversity of the filtered list of *Salmonella* genomes. The relative size of each bubble indicates the relative number of genomes reported to belong to that serovar. Each bubble is colourized based on the number of unique 330 gene cgMLST profiles, which were found within a given serovar. Darker bubbles indicate a higher number of unique cgMLST profiles.

**Fig. 3.**
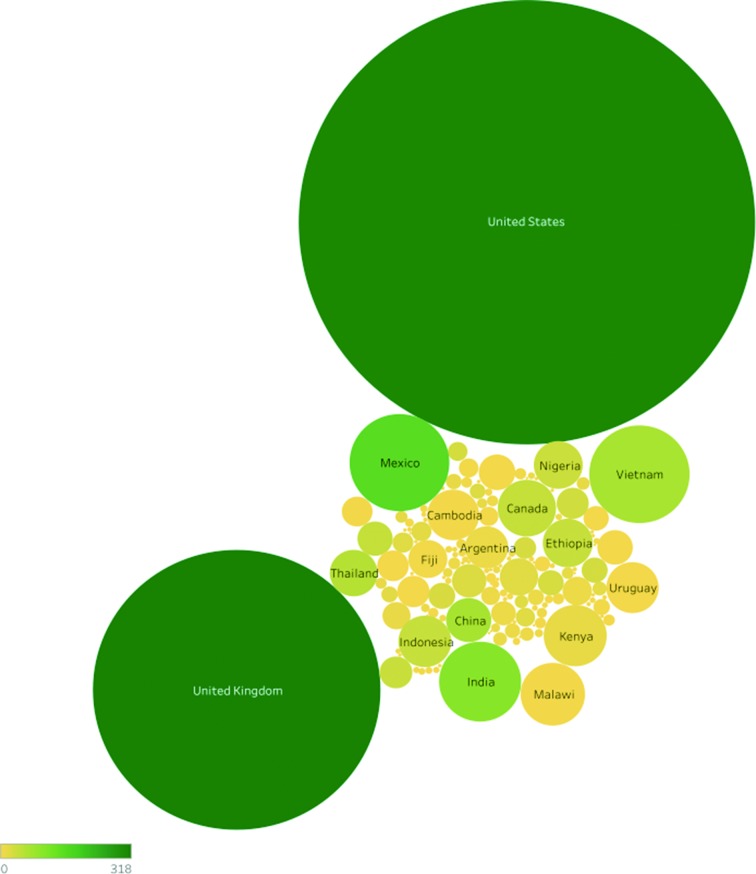
Bubble graphs highlighting the bias in the SRA dataset to the USA and UK due to their large surveillance programmes. The size of the bubble indicates the relative number of genomes listing that country as their collection source and the colour of the bubble indicates the number of distinct serovars reported from that country.

A total of 42 400 *Salmonella* Illumina paired-end samples passed the genome quality assessment filters. Using the cleaned and standardized sample metadata, the serovar composition of the SRA was determined and is highlighted in Fig. 2. Three serovars, Typhimurium, Enteritidis and Typhi, account for more than 60 % of the genomes in the SRA (Fig. 2). Given that Typhimurium and Enteritidis are cosmopolitan serovars that are in the top five reported serovars globally, it stands to reason that they should represent a large fraction of the database. Within each serovar, the number of unique cgMLST profiles grows as more samples are collected for a given serovar (Fig. 2). The data in the SRA come from 142 countries with the majority of the dataset coming from the USA and UK (Fig. 3). Both the USA and UK sequencing programmes have deposited sequences from diverse *Salmonella* serovars (Fig. 3) but as stated above, there is a bias towards the submission of common serovars. Unfortunately, many SRA records are deposited with limited sample information, which reduces the value of the records. Approximately 13 % of all records have no metadata to contextualize the sample and a total of 30 % lack serovar information (Data S1). The initial set of 20 446 genomes lacking serotype information were quality assessed and 19 557 were of sufficient quality that SISTR was able to produce a reliable and unambiguous serovar prediction for 17 728 (Data S1). These *in silico* serotyped genomes can now be used as part of reference databases for *Salmonella* whereas previously they would be treated as genomes of undefined serovar.

### MLST accuracy

Serotyping prediction from *in silico* MLST of WGS data has been proposed due to the high degree of association between specific STs and traditional serovars [3, 8]. The seven MLST loci were readily retrievable, with all 42 400 genome assemblies possessing the complete complement of seven loci and 2219 having incomplete, novel or multiple alleles for a given gene (Table 2). Of the genomes with the complete complement of loci, there were 2289 that could not be assigned to a known serovar (Data S1) due to no known serovar assignment to that ST.

**Tables 2.** In total, 42 400 draft genomes, which passed all of the quality criteria from Table 1, were examined for the concordance of *in silico* serovar predictions with the reported serovar Each genome was categorized into one of five different categories according to the criteria established in the Methods.

Category	SISTR	MLST
Type 0: Full match	38 954	36 954
Type 1: Incorrect reported serovar	2115	1804
Type 2: Serovar variant detected	1305	891
Type 3: Incorrect predicted serovar	26	462
Type 4: Untypeable	0	2289

An analysis of the reference MLST database (http://mlst.warwick.ac.uk/) shows that in some cases there are errors in the serovar assignment within STs or that the genetic resolution provided by MLST is insufficient to distinguish them (Data S2). Of 1661 STs with an assigned serovar there were 21 that contained two different subspecies (Data S2). On further inspection, there are 122 STs that contained two or more distinct serovars (Data S2). Notably, ST 11 contains isolates from the serovars Antarctica, Dublin, Enteritidis, Moscow, Nitra, Rosenberg and Typhimurium, with ~97 % of isolates in the cluster belonging to Enteritidis (Data S2). Based on the data available, it is not possible to determine whether the serovar assignments are correct; in the case of Nitra it is known that this serovar is difficult to distinguish from Enteritidis.

### Comparison of MLST and SISTR serovar predictions with reported serovars

We examined the concordance between the reported serovar provided with the record and the prediction produced by MLST and SISTR. Within the Type 0 category (i.e. Full match), SISTR produced concordant predictions for 91.91 % of the dataset with MLST performing slightly lower at 87.51 %. Both methods perform similarly for Type 1 (i.e. Incorrect reported serovar) and Type 2 (i.e. Serovar variant detected) (Table 2). MLST does result in 451 more errors of Type 3 (i.e. Incorrect predicted serovar), where MLST has predicted a different serovar from SISTR and from what is reported on the record (Table 2). Since genotypic tests do not always correlate with phenotype and the general tendency of some individuals to merge mono- and bi-phasic serovars into one, the overall accuracy of the different tools can be calculated by combining the Type 0 and Type 2 categories to get an overall concordance of 94.98 % for SISTR and 89.25 % for MLST. These numbers are in keeping with what has been reported previously for the two tools [8, 17]. MLST could not produce a serovar for 2289 of the 42 400 genomes, which represents 5.4 % of genome MLST queries, despite the availability of antigenic information in the WGS data.

### In-depth analysis of serovar Oranienburg and Othmarschen genomes

**Fig. 4.**
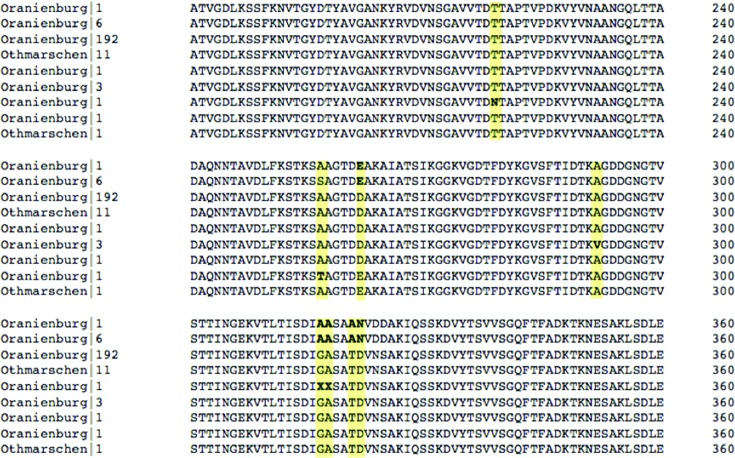
Multiple sequence alignment of Oranienburg and Othmarschen FliC protein sequences with conserved ends removed. Amino acid variants are in bold and highlighted in yellow. Sequence labels consist of the reported serovar and the number of times that unique FliC protein was seen within that serovar.

**Fig. 5.**
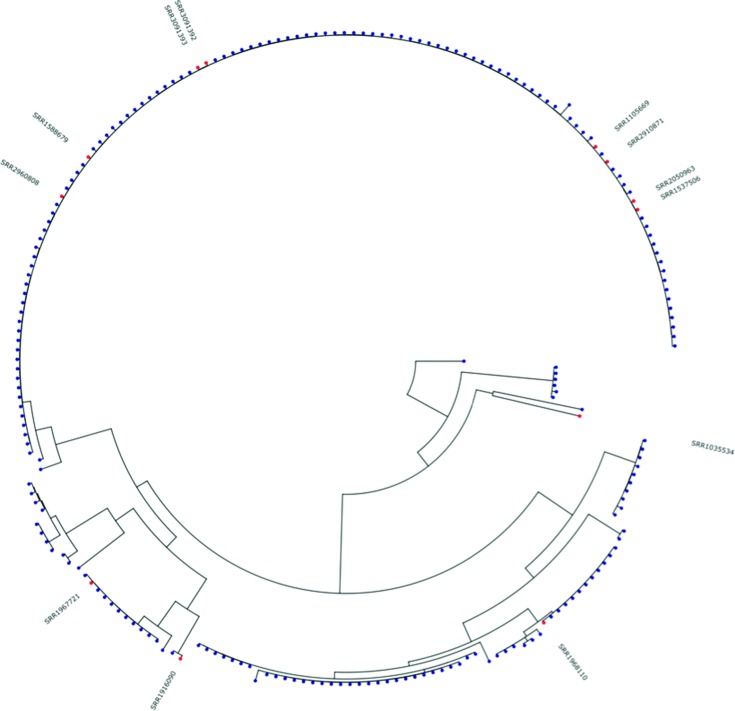
Core genome SNP tree produced by Parsnp alignment of *de novo* assemblies of isolates which were reported to be Oranienburg or Othmarschen, and where SISTR confirmed that their antigenic formulas were accurate. Othmarschen isolates are highlighted in red.

Due to the limited number of representative genomes for some serovars, it is not always possible to determine whether the serovar predictions are correct when there is a conflict with the reported serovar. The 330 genes utilized in the SISTR cgMLST scheme provide an approximation of the genetic distance between serovars and although this is based on <10 % of the genome, this approximation is useful for disambiguating serovars with similar antigenic formula. There are, however, several cases where the reported and predicted serovars cannot be consistently resolved. In most cases, one serovar is far more prevalent than another, so the rare serovar is less likely to be the true serovar. An example of this is observed with serovars Oranienburg (6,7,14:m,t:-) and Othmarschen (6,7,14:g,m,t:-). The *fliC* gene in both cases shows nearly identical sequences with no consistent amino acid differences between isolates of each serovar (Fig. 4). To determine if the Othmarschen genomes cluster separately from Oranienburg, a core genome SNP tree was produced using Parsnp from all of the isolates reported as either serovar and possessing the antigenic formula 6,7,14:m,t:- and of subspecies designation *enterica*. The Othmarschen isolates do not form a consistent grouping within the tree (Fig. 5), suggesting that these isolates may be incorrectly classified.

## Discussion

The benefits of a globally distributed sequencing network for foodborne infections are undeniable but problems of standardization and quality of the data need to be addressed. However, there has not been a comprehensive assessment of the quality of WGS data for pathogens such as *Salmonella* in public sequence repositories. This is concerning because errors in identification will propagate as the sequence data are used to identify unknown genomes. Furthermore, wrong or missing metadata greatly depreciate the value of the genomic data and the usefulness of WGS to identify multi-year and multi-state outbreaks [19, 20].

The problems currently faced by the public health and food safety communities have been a challenge for the DNA barcoding and microbial diversity communities for many years. With over 2 million *cytochrome oxidase I* and 13 million 16S nucleotide records in GenBank, there is a great need for verified records. Faced with large volumes of data, there were inevitably errors introduced to the database either within the DNA sequence itself (chimeras, pseudogenes, contaminations, poor quality chromatograms) or within the metadata (identification, location, host, isolation source) [11, 12, 15, 21–23]. The proliferation of errors within the database prompted action on the part of the communities to institute quality control mechanisms.

Errors in public repositories are inevitable given the volume of data being generated in near real time, which means that data validation and curation should be integrated into the workflows of laboratories submitting data to public repositories. We find that more than a quarter of the genomes examined for this study contain no metadata other than the submitter of the isolate (Data S1). This paucity of metadata can limit the usefulness of the genome sequence data and limits its use for research questions, also reducing the ability to discover errors in the data. In the era of WGS there is still a great need for serovar information since this classification has formed the basis for surveillance and epidemiological programmes for nearly 100 years. Loss of this historical information would disrupt existing surveillance programmes as it will take time for the global community to fully adopt WGS. Given the importance of serovar information for classifying *Salmonella* isolates, it is concerning that 20 446 out of 67 758 genomes did not include this critical piece of information. While this can probably be overlooked for the highly common serovars, it dilutes the strength of WGS as a tool in public health genomics because it limits access to data such as ‘reported serovar’, geographical location and date/time of isolation that are used in outbreak investigations, surveillance tracking and phylogenetic studies to complement genomic analyses. We have predicted the serovars for these 20 446 genomes using SISTR and made this information available to the scientific community (Data S1) so as to improve the usability of a considerable amount of *Salmonella* genomic data in the public domain. During the course of this study, we also observed a lack of standardization in the representation of serovar names because the serovar field is free text, which poses a challenge for comparing isolates. For example, the serovar Enteritidis has no additional variants but we observed four different descriptions in the SRA: Enteritidis, Enteriditis, Enteritidis atypical and *Salmonella* Enteritidis. The problem is worse for serovars that have multiple reported variants such as Typhimurium (Data S1).

Public Health England (PHE) has recently replaced routine serological testing with WGS and serovar prediction using MLST in 2015 [8]. MLST is based on the principle of indexing genomic variation of seven housekeeping genes to provide an ST identifier, which correlates strongly with serovar [3, 24]. Similar to the findings of Yachison *et al.* [2] (88.3 %), we find that MLST predicts serovar with 89.25 % concordance to the reported serovar. This concordance demonstrates that MLST does correlate well with serotype across very different sized datasets. A drawback to MLST inference of serovar information is that novel STs cannot be assigned to a serovar without traditional serotyping and we found a substantial number of genomes that could not be assigned to known STs with an associated serovar. Serovar prediction of an isolate from WGS data using similarity searches of the genes responsible for the serological phenotype has been implemented in SeqSero [25]. The drawback of using this approach is that multiple serovars can have the same antigenic formula when considered at the serogroup level. Additional complications include that the genetic bases of a phenotype may be outside the *rfb* locus for the O antigen and that the presence of a flagellar gene does not mean that it will be expressed. The performance of SeqSero was also investigated by Yachison *et al.* [2] and they found that its performance was similar to MLST at 88.2 % concordance with traditional serotyping [2].

The SISTR was developed to combine the strengths of cgMLST and antigen detection to provide highly accurate serovar predictions and refine predictions where the antigenic formula was ambiguous [17]. The concordance between reported and predicted serovar increases slightly when using SISTR, which reports that 94.98 % of records matched the reported serovar. The amount of discordance between reported and predicted serovar found is within the range that was previously reported [8, 17] and probably represents the amount of processing and identification errors in the database. In 69 % of discordant records, the genetic evidence clearly indicated that the serovar was incorrectly specified in the SRA record (Data S1). The next largest category of discordant calls, ~28 % of errors, was the misreporting of monophasic serovar variants such as Typhimurium and I 1,4,[5],12:i:- (Data S1). Examination of the records in question shows that many of these discordant records were from a small number of projects, so it is likely that errors were propagated during entry of the specimen data into the SRA (Data S1).

In the past, accuracy estimations of *in silico* serovar prediction tools have been made on different sets of isolates [3, 8, 17, 25] which complicates comparisons between tools. As public health moves forward with *in silico* predictions of serovar, it is becoming increasingly apparent that in addition to reporting the serovar, the method for deriving the serovar should be reported because of the strengths and weaknesses of the various approaches. The list of isolates here marked as concordant can serve as a standard set of high-quality genomes against which new prediction tools for *Salmonella* can be assessed. This will allow equal comparisons of new tools against a known benchmark of sufficient size and diversity to truly test new tools.

cgMLST is the natural successor to MLST by extending the principle of indexed genetic variation to hundreds and even thousands of loci [26, 27]. The cgMLST schema implemented in SISTR consists of 330 loci originally selected on the basis of robust performance on genome sequences representing widely varying sequencing quality metrics. Results from this large-scale validation are consistent with this design, as they were readily retrievable from genome assemblies representing a wide variety of serovars and genetic backgrounds and can provide a useful estimate of genetic distance between two isolates. With the exception of a few very closely related serovars such as Enteritidis/Nitra and Oranienburg/Othmarschen, isolates from a given cgMLST cluster shared the same single serovar (Data S1). Comparatively, MLST yields several predominant clusters (i.e. STs), which consist of isolates from multiple serovars. Thus, serovar predictions using MLST alone can result in merging of several serovars into a single cluster, which will impact reporting of serovar prevalence (Data S1).

With thousands of sequencing experiments being deposited monthly into the public archives, sequence data must be paired with high-quality metadata to ensure that the massive amount of data being produced will have value to the research and diagnostic communities. The minimum information about a genome sequence (MIGS) specification which has existed since 2008 [28] was developed by the Genomics Standards Consortium but less than 49 % of isolates tested in this study fulfil these requirements. Submitters must make improvements to both the quality and the quantity of metadata available for sequencing experiments deposited in public repositories. In conjunction with improved metadata standards, we strongly recommend NCBI implements routine subtyping of isolates for clinically relevant pathogens such as *Salmonella* where species information is not sufficiently informative. The validated collection of genomic metadata presented here improves the value of the records in the SRA by providing reliable serovar information from SISTR alongside standardized metadata. Unfortunately, NCBI does not support third-party annotations or assembly of data, but platforms such as EnteroBase (http://enterobase.warwick.ac.uk/) could serve as repositories where the community can add and improve metadata to the existing records without this restriction.

### Conclusions

WGS databases such as the SRA from NCBI allow access to genomic information to anyone around the world without cost and without restriction. It is noteworthy that institutions such as the Centers for Disease Control and Prevention (CDC), Public Health England (PHE), US Food and Drug Administration (FDA) and others have been pioneers in depositing large volumes of WGS data from routine surveillance and outbreaks into the SRA. These early adopters have provided proof of concept on the value of a public data paradigm. The data produced by WGS are readily exchanged and utilized by researchers and public health professionals to address their specific research questions. The falling cost and increased throughput of WGS has resulted in an explosion of data but there has been little thought given to the trustworthiness and value of any given record in the database. As the use of WGS increasingly becomes routine practice for the identification and characterization of pathogens such as *Salmonella*, the need for data standards and curation will only increase. Errors in these data should be of great concern because they can impact the public health response to pathogens such as *Salmonella.*

For *Salmonella*, we recommend that the quality filters described here are sufficient for having high confidence in serovar assignment predicted from assemblies. However, the sequencing quality criteria will change for different applications where considerations must be given to the depth of coverage and not just the final consensus. SISTR is a powerful tool, which can accurately predict serovar from draft genomes and can discover problems in serovar identification. The presented fully validated dataset of 67 758 genomes (Data S1) with standardized metadata and full serotype information that we have produced during the course of this study will be a valuable asset for users of the genomic data in the public health and food safety community.

## Data bibliography

1. Processed metadata for SRA isolates used in this manuscript. Information on geographical location, collection date, source, serovar and assembly metrics are included. James Robertson, Catherine Yoshida, Peter Kruczkiewicz, Celine Nadon, Anil Nichani, Eduardo N. Taboada, John H. E. Nash. FigShare. DOI: 10.6084/m9.figshare.5464396. https://figshare.com/s/fc4adaac52a678d92c8b (2017).
2. MLST information was downloaded from http://mlst.warwick.ac.uk/mlst for *Salmonella*. James Robertson, Catherine Yoshida, Peter Kruczkiewicz, Celine Nadon, Anil Nichani, Eduardo N. Taboada, John H. E. Nash. FigShare https://figshare.com/s/fc4adaac52a678d92c8b (2017).

## Supplementary Data

41180 Supplementary File 1

## Supplementary Data

663 Supplementary File 2
